# Defining a role for hemoglobin in Parkinson’s disease

**DOI:** 10.1038/npjparkd.2016.21

**Published:** 2016-10-20

**Authors:** James Freed, Lisa Chakrabarti

**Affiliations:** 1 School of Veterinary Medicine and Science, University of Nottingham, Sutton, Bonington, UK

## Abstract

Hemoglobin (Hb) has been shown to be expressed within dopaminergic neurons and to have a role in maintaining iron and mitochondrial homeostasis. In Parkinson’s disease tissues, Hb has been localized to the mitochondrion. Though heme synthesis occurs within the mitochondria, the localization of Hb to this organelle has only recently been described. It is now important to understand whether Hb expression is protective or is a part of the neurodegenerative process. It is possible that the accumulation of neuronal or mitochondrial Hb is initially protective, but over many decades causes pathology. Studying Hb in neurons can give insight into the iron accumulation seen in the brain and the potential role of alpha-synuclein as a ferrireductase. In this review, we discuss the interactions of neuronal and mitochondrial Hb with other proteins and its possible role in pathways relevant to Parkinson’s disease.

## Parkinson’s disease (PD) is a neurodegenerative disorder, affecting more than 5 million people worldwide

There have been many breakthroughs characterizing this disease, yet PD remains difficult to treat for the entirety of the disease course.^1^ There are specific molecular pathways that appear particularly promising in terms of therapeutic targeting, for example understanding alpha-synuclein (α-syn) or the leucine-rich repeat kinase 2. There are also consistent pathological signs such as Lewy bodies and iron accumulation. Much more recently, imaging technology efforts have revealed a “swallow tail” hallmark that is proposed to change in PD.^2^ It is always hoped that newer technologies can produce better solutions and markers for PD. This neurodegeneration has a recognizable presentation, which can be apparent to the trained neurologist allowed consistent follow-up. Yet there is no molecular or biochemical test of peripheral tissues, which means that cases can only be confirmed at postmortem by the loss of dopaminergic neurons in the substantia nigra pars compacta and observed intraneuronal α-syn aggregates. Dopaminergic neurons are considered particularly prone to oxidative stress because of dopamine metabolism, increased iron levels, decreased glutathione levels, calcium entry, and mitochondrial dysfunction.^3,4^

## Hb and parkinson’s disease

Recently, Hb RNA and protein expression have been measured within dopaminergic neurons in mouse, rat, and human brain.^5–10^ Our group has observed changes in mitochondrial Hb in PD and also in ageing mice.^8^ Adult human Hb is a heterotetrameric protein composed of two α- and two β-subunits. Each subunit of Hb contains a heme group with a central Fe^2+^ ion, allowing Hb to bind to gaseous molecules. Hb’s primary function is considered to be the transportation of oxygen, carbon dioxide, and nitric oxide within the blood, to maintain the tissues of the body.^11^ Through gaseous transport and exchange, this molecule also performs an important buffering role. There is gathering evidence that Hb levels within the brain affect the risk of developing neurodegenerative diseases including PD.^11–15^ Reports associate Hb and neuronal Hb levels with Alzheimer’s disease, dementia with Lewy bodies, age-associated cognitive decline, and argyrophilic grain disease.^10^ We postulate here that Hb has an intraneuronal role in contributing to the changes in oxidative status and mitochondrial dysfunction that have been associated with PD.

## Heme synthesis occurs within the mitochondria

An important question to ask when a molecule is suggested to be involved in a disease process is which part of the cell it inhabits. Hb is formed from the globin protein together with the iron-containing heme moiety. Heme is a heterocyclic macromolecule comprised of four pyrrole rings and a central ferrous ion, its structure allows it to reversibly bind to gaseous molecules such as oxygen, nitric oxide (NO), and carbon monoxide (CO). Heme is incorporated in several protein groups including the cytochrome family, Hb, peroxidase, and nitric oxide synthase.^16^ The tetrapyrrole ring of heme (Protoporphyrin IX) is synthesized within the matrix of the mitochondria, from the metabolism of glycine and succinyl-CoA—the Fe^2+^ ion is then inserted into the center of protoporphyrin IX via ferrochelatase to form heme.^16,17^ Several carrier proteins have been identified to relocate heme from the mitochondrial matrix into the cytoplasm.^18,19^ It has been demonstrated that heme is present within neurons, and also that heme synthesis declines with age.^20,21^ Decreased heme biosynthesis in non-erythroid cells causes mitochondrial complex IV inhibition, oxidative stress, iron accumulation, and cell death.^17^ Overall, heme and also Hb are intimately associated with the mitochondrion. Expression in neuronal populations allows us to examine the possibility that Hb is indeed important in neuronal mitochondria. With evidence gathered in favor of this location, it also makes very good sense to have the protein responsible for oxygen transport in the vicinity of the very organelle that utilizes oxygen to generate adenosine tri-phosphate (ATP).

## Connecting Hb degradation to PD pathology

Eighty per cent of heme degradation has Hb as the source molecule.^22^ Oxidative stress induces the release of heme from Hb.^19,23^ Heme oxygenase-1 catalyzes the rate-limiting step of heme breakdown,^24^ which has been shown to accelerate the oxidative neurotoxicity of Hb in cortical neurons.^25,26^ Interestingly, heme catabolism by heme oxygenase-1 also inhibits cyclooxygenase-2 expression and prostaglandin synthesis.^27^ It has been demonstrated that increased expression of cyclooxygenase-2 in brain dopaminergic neurons is implicated in neuronal inflammation.^28,29^ Overexpression of heme oxygenase-1 in the substantia nigra (SN) of 1-methyl-4-phenyl-1, 2, 3, 6-tetrahydropyridine (MPTP)-intoxicated mice significantly increased the survival of dopaminergic neurons.^30^ On the basis of these findings, it could be suggested that the catabolism of heme via heme oxygenase-1 is a means of decreasing cyclooxygenase-2 expression to facilitate neuroprotection.

Heme degradation from oxygenated Hb leads to the formation of superoxide (O_2_^−^), which is subsequently converted into hydrogen peroxide (H_2_O_2_) via superoxide dismutase.^22^ It has been demonstrated that ROS such as peroxide denature Hb and induce the release of iron from heme.^3,31^ It could be postulated that ubiquitin could then conjugate to damaged Hb, targeting it for degradation.^32^ Excess levels of Hb might therefore contribute to oxidative stress and iron accumulation in neurons, resulting in a deleterious cycle of heme breakdown, oxidative stress, Hb denaturation, and over time—neurodegeneration. This pathway is further supported by a study describing intraventricular injections of Hb in neonatal rats resulting in decreased hippocampal volume and neuronal degeneration.^33^

## Hb levels change in ageing and neurodegeneration

Hb levels within the brain tend to decrease with ageing, the cause of this remains unknown.^12,34^ Perhaps the levels in the brain mirror that of systemic Hb, which can also be seen to decrease in older individuals.^35^ This could be due to chronic levels of inflammation or a reduced functionality of bone marrow. It may be that due to an increased breakdown of the Hb protein, there is a release of heme that then contributes to the build-up of brain iron. Double labeling immunofluorescence and confocal microscopy demonstrate reduced levels of Hb α- and β-chains in 80% of dopaminergic neurons with α-syn deposits and Lewy bodies in the SN and medulla oblongata of postmortem PD patients.^11^ In addition, it has been demonstrated that Hb mRNA levels are downregulated in rotenone-treated rodent nigrostriatal dopaminergic neurons where complex 1 is inhibited to produce a model of PD without neuronal death.^6^ However, although Hb levels tend to decrease with ageing, men who maintained higher levels of Hb were found to have an increased risk of developing PD.^12^ These observations may lend weight to the idea that increased degradation of Hb is causing the pathology evidenced by increased brain iron. In individuals with non-limiting Hb levels, the Hb breakdown pathway may be left uncontrolled or constantly active. Over many decades, this could lead to the pathology observed. In summary, we suggest that individuals with abnormally high neuronal Hb levels could be particularly vulnerable to development of neurodegeneration.

## Hb and mitochondrial homeostasis

The function of Hb in the central nervous system remains unclear, but there is evidence that it has a role in mitochondrial homeostasis. Hb has several roles in nonerythroid cells, such as regulating iron metabolism and transporting oxygen to the mitochondria in order to accept electrons at the end of the respiratory chain. Hb has also been shown to regulate genes involved in oxygen homeostasis and oxidative phosphorylation, linking Hb expression to mitochondrial activity.^10^ Hb overexpression in mouse dopaminergic cell lines upregulates the expression of genes involved in mitochondrial oxidative phosphorylation.^11^ Conversely, administration of rotenone (a well-known complex I inhibitor) downregulates Hb α- and β-chain expression in rat nigral, striatal, and cortical neurons.^11^ We suggest that a reduction in neuronal Hb in PD patients could contribute to an imbalance in mitochondrial homeostasis and hypoxia. This would also result in an increase in reactive oxygen species production. For example, superoxide (O^2^^−^) is produced from mitochondrial complexes I and III of the electron transport chain, which can then react with other by-products to form hydroxyl radicals (OH^−^). Accumulation of free oxygen radicals (oxidative stress) can damage the mtDNA, essentially leading to mitochondrial dysfunction and neuronal cell death.^36^ PD brain mitochondrial fractions show a significant loss of Hb proteins from the organelles into the surrounding cytoplasm.^37^ It has been demonstrated that mitochondrial and Hb proteins co-localize in affected areas of the substantia nigra.^9^ Future studies could focus on the impact that decreased Hb levels have on mitochondrial electron transport chain activity.

## Hb forms a complex with α-synuclein, reducing levels of neuronal Hb

Alpha-syn aggregates and Lewy body formations are pathological hallmarks of PD.^1^ Alpha-syn can impair mitochondria, enhance oxidative stress, and disrupt iron homeostasis.^38^ The α-syn protein has also been assigned ferrireductase properties, indicating a significant role in iron homeostasis.^39^ In ageing brains, neuronal Hb levels and mitochondrial function are greatly reduced in neurons that accumulate α-syn. Very recent work demonstrates that α-syn and neuronal Hb can form a complex in brain tissue in ageing cynomolgus monkeys.^40^ *In vitro* studies using a cultured dopaminergic cell line show that intracellular accumulation of α-syn causes an elevation in nHb-α-syn complex levels in both mitochondrial and cytoplasmic fractions as well as a reduction in mitochondrial free nHb. Yang *et al.* suggest that increased intracellular α-syn reduces the pool of free nHb in mitochondria as a result of nHb-α-syn complex formation. This is consistent with Hb-forming protein aggregates identifiable in postmortem brains of PD patients.^10^ Of course when we discuss iron or Hb regulation, it is important to bear in mind that males and females have different methods for managing iron during their lifespan. It is interesting that males have no active mechanism to actively remove iron from their bodies during their lifetime and are also more likely to be diagnosed with PD.^37,41,42^ We find differences in mitochondrial Hb in comparisons of male and female PD brains.^37^ Our data set compared age-matched postmortem brain mitochondrial hemoglobin ratios that we found to decrease with disease duration in female cerebellum mitochondria with a less discernible effect in male cerebellum. Sub-mitochondrial localization of hemoglobin also appeared to be different between males and females with females apparently maintaining a closer to normal distribution of mitochondrial hemoglobin across the organelle. On the basis of these findings, we suggest that Hb and iron accumulation and regulation in PD should always be considered within the context of gender.

## Neuronal Hb may contribute to elevated iron levels in the substantia nigra

Iron is found in several proteins that are vital for mitochondrial respiration and oxygen transport, such as Hb and iron–sulphur clusters within the inner membrane of the mitochondria.^27,43^ Hb is the most significant source of peripheral iron in humans, indeed most of the iron in the body is recycled from senescent erythrocytes.^12,27,43^ Cytosolic Fe^2+^ is utilized for purposes such as heme synthesis within the mitochondrion and dopamine synthesis via tyrosine hydroxylase.^43,44^

Iron accumulation within the substantia nigra occurs with ageing.^45,46^ In patients with PD, this effect has been found to be further magnified to over 30% higher than controls combined with a shift in the iron II/II balance toward iron III in severely affected PD substantia nigra.^47–49^ Of particular relevance to PD is the presence of the iron-containing pigment, neuromelanin. Neuromelanin is normally found to accumulate with age in neurons of the substantia nigra, giving them their characteristic dark color.^45,50^ The highly selective loss of the pigmented neurons is a pathological hallmark of PD.^50^ High levels of free iron within the brain can become cytotoxic, promoting oxidative stress, mitochondrial protein dysfunction, α-syn aggregation, and neuronal cell death.^27,46,50,51^Therefore, the dysregulation of iron metabolism would be expected to have a negative impact on mitochondrial respiration and potentially lead to chronic oxidative stress.^43^ It is not yet fully understood whether iron accumulation is a primary or secondary event in age-related neuronal cell death.^43^ However, evidence suggests that iron accumulation is an early event in dopaminergic cell loss.^3^ The link between Hb levels and iron accumulation within the brain is yet to be fully assessed.

### Conclusion

It has been shown that Hb levels are affected with ageing and neurodegeneration. However, many questions remained unsolved as to whether or not Hb levels contribute to the cellular processes observed in neurodegeneration, such as increased iron levels, decreased mitochondrial activity, and increased oxidative stress within the brain. Further studies could focus on comparing the differences in heme synthesis between ageing and neurodegeneration in order to establish whether cytotoxic heme levels correlate with loss of dopaminergic neurons in PD. Is it a reduction in heme biosynthesis or an increase in heme degradation that elevates the risk of developing PD? As heme breakdown could be contributing to increased iron accumulation and ROS production, determining the balance of heme production versus degradation is likely to be crucial. Accumulation of iron within the brain can be contributed to the breakdown of heme from neuronal Hb. Increased oxidative stress and reduced mitochondrial activity can also be explained by insufficient transport of oxygen to the mitochondria within neurons. However, it is still to be determined whether neuronal Hb is the main source of oxygen delivery to neurons. The identification of α-syn-neuronal Hb complexes in brain tissues allows us to consider exploring α-syn function as the regulator maintaining equilibrium. Iron accumulation, oxidative stress, mitochondrial dysfunction, and α-syn complex formation are all principal players in PD. From the evidence collected here, we propose that Hb could have an important role in the processes that culminate in PD neurodegeneration.
